# Experimental and Numerical Studies of Two- and Three-Layer Corrugated Boards in Bending Test

**DOI:** 10.3390/ma18184351

**Published:** 2025-09-17

**Authors:** Gabriela Kmita-Fudalej, Leszek Czechowski

**Affiliations:** 1 Centre of Papermaking and Printing, Lodz University of Technology, 93-005 Lodz, Poland; 2 Department of Strength of Materials, Lodz University of Technology, 90-537 Lodz, Poland; leszek.czechowski@p.lodz.pl

**Keywords:** stiffness of paperboard, bending tests, mechanical properties

## Abstract

This paper deals with the analysis of four-point bending two- and three-layer corrugated boards along the direction perpendicular to the machine direction. The taken segments of paperboard were examined to determine the bending stiffness for three different configurations. The investigations were carried out experimentally and numerically. The tests of bending were analysed only in the elastic range of the material. Each configuration of paperboard was modelled as an orthotropic material. The numerical analysis was based on the finite element method by applying Ansys^®^ software. Several material properties and the thicknesses of papers were assumed to determine the general stiffness in bending. In the analysis, two different discrete models based on geometries of the paperboard were elaborated to adjust the results to the experimental ones. The results of analyses for some configurations showed good agreement with the experiment. This paper indicates some differences in stiffness between two- and three-layer paperboards.

## 1. Introduction

Corrugated board is one of the most popular packaging materials in the world, valued for its strength, versatility, and sustainability. Packaging made from corrugated paperboard has many advantages over other packaging materials, such as plastic, metal, and wood. Nowadays, some very important advantages are its low production costs and eco-friendliness and biodegradability, as well as its recyclability and reusability [1,2,3,4,5]. Corrugated board boxes are lightweight, which has an impact on their ease of use and does not significantly increase the weight of the goods being transported. Packaging has become an integral part of the distribution of many goods, providing a link between manufacturers and final purchasers. Thanks to the good strength properties of corrugated board, boxes protect packaged goods during transport and storage. Packages are being designed to protect products from failure before they are delivered to customers. Due to the increasing number of online orders, reliable and cost-effective packaging solutions for shipping goods are essential, which entails an increase in packaging consumption in the e-commerce industry. The static pressure resistance of *BCT* boxes and other strength properties play an important role in the distribution of almost everything we buy and consume, and have been the focus of corrugated board research as a construction material since its production began [6,7]. Based on the literature, there are many works related to the strength and stiffness of papers or paperboard. The authors of the paper [8] relied upon mathematical models to predict the stress peaks of corrugated paperboards. Bai et al. [9] analysed the strength of a single corrugated board subjected to axial crushing. Fadiji et al. [10,11,12] investigated the strength and creep of a paperboard box due to compression in different conditions. In the article [13], the results of measurements of the static pressure resistance of *BCT* flap boxes with contacting flaps, as well as the conditions of the measurements using two measuring instruments, were analysed. The first gauge had both load plates fixed rigidly, while the second gauge had the lower plate fixed rigidly and the upper plate fixed on a swivel. Wallmeier et al. [14] analysed the deep drawing of paperboard. Results of numerical experiments based on corrugated board strength were contained in works [15,16,17,18]. Zaheer et al. [19], by using the finite element method, studied the strength of a compressed paperboard box. The analytical and numerical approaches to determining the strength of paper tubes subjected to lateral load were considered in the papers [20,21]. The authors of the paper [22] analysed the strength of three paperboards. Hämäläinen et al. [23] studied the transverse shear properties of paper using a short span compression test. Considering the first-order shear deformation theory, Hernández-Pérez et al. [24] analysed the twist stiffness of single and double-wall corrugated board. Other results referring to the honeycomb board’s strength were included in the papers [25,26,27,28]. Mentrasti et al. analysed experimentally and analytically the behaviour of creased paperboard under large bending in papers [29,30]. Bolzon and Talassi [31] investigated the strength of anisotropic paperboard composites up to their damage threshold using the burst strength testers. The paper [32] included the results of the flexural damage of honeycomb paperboard based on FEM and experiment. Minh [33] presented an analysis of an analytic homogenization model for a five-layer corrugated board subjected to transverse loading. On the other hand, the authors of the paper [34] conducted tests of the crushing of five-layer corrugated board. Han et al. [35] explored the strength of bonded corrugated sandwich beams using a three-point bending test. Analysis of buckling and post-buckling of composite thin-walled structures has been carried out in several studies [36,37,38,39,40]. Park et al. [41] conducted bending stiffness tests on three-layer and five-layer corrugated boards with different geometric parameters using finite element (FEM) simulation, and also measured bending stiffness using the four-point bending method for comparison. They obtained similar results for the force–deflection relationships obtained during the measurements with the results obtained by finite element (FEM) simulation in the cross direction (*CD*) of the corrugated boards tested. However, it was not possible to simulate the bending stiffness in the machine direction (*MD*) using FEM simulation. Extensive research on three-layer C-wave corrugated board and boxes made from it is presented in the article by Cillie and Coetzee [42]. Both experimental tests and finite element (FE) simulations of deformations in the corrugated board plane were carried out. The corrugated board was modelled using nonlinear FEM with a linear material model. Based on the research conducted, the authors observed that initial defects have a significant impact on the accuracy of the models. Taking defects into account and adopting nonlinear material models could improve the accuracy of the models. In the article by Russo et al. [43], simulations were also carried out using finite element analysis and *ECT* measurements of three-layer corrugated board and *BCT* boxes made from it. The tested corrugated boards were made of various fibrous raw materials and had E, B, and C waves. The simulations aimed to predict the results of *ECT* and *BCT* tests and the impact of different flutes and materials on the load–displacement curves. Metar and Fitas [44] performed a finite element analysis of corrugated boards subjected to flat compression using FEA software. They used three-layer corrugated boards with A, B, and E waves for their research. They analysed the distribution of pressure on the waves along the top and bottom layers. They compared the load–deflection curves obtained using the models with the curves obtained through measurements.

The aforementioned papers concern the strength/stiffness, especially of three-layer and five-layer corrugated boards with A, B, C, and E waves. The height of the waves in the tested corrugated boards ranged from 1.2 mm to 5 mm, which are the most popular waves used in corrugated boards in the packaging industry. Two-layer corrugated board is not a popular material used in research, nor is two- and three-layer board with an S-wave with a wave height of 8.6 mm, which gives the work a scientific character and brings elements of novelty to the scientific field. The present investigation deals with experimental and numerical analysis of corrugated paperboard subjected to a four-point flexural test where cross directions cover the bending plane. Experimental tests were carried out for different thicknesses of flat-layer and of corrugated paper with two or three layers to analyse the stiffness. Two types of numerical models (without homogenization) were considered to adjust to experimental results. The simulations were performed in Ansys^®^ version 2025 R1 [45] for large displacements on the basis of Green–Lagrange equations. Comparison of 2-layer paperboard with 3-layer paperboard can be beneficial to design advanced structures made of paperboard.

## 2. Problem Formulation

This chapter is divided into five subsections. The first subsection describes the material tested and the parameters for measuring the bending stiffness (*BS*). The second subsection describes the methodology for measuring and calculating the physical properties of the papers used to produce the tested corrugated boards and the method for measuring the geometric parameters of the corrugated boards. The fourth subsection contains the method for measuring the bending stiffness (*BS*) and a description of the instrument used. The next two solutions concern the methodology for calculating the *BS*, the analysis of the *BS* results, and the construction of the model used in the FE simulations.

### 2.1. Objects of Analysis

**Figure 1:**
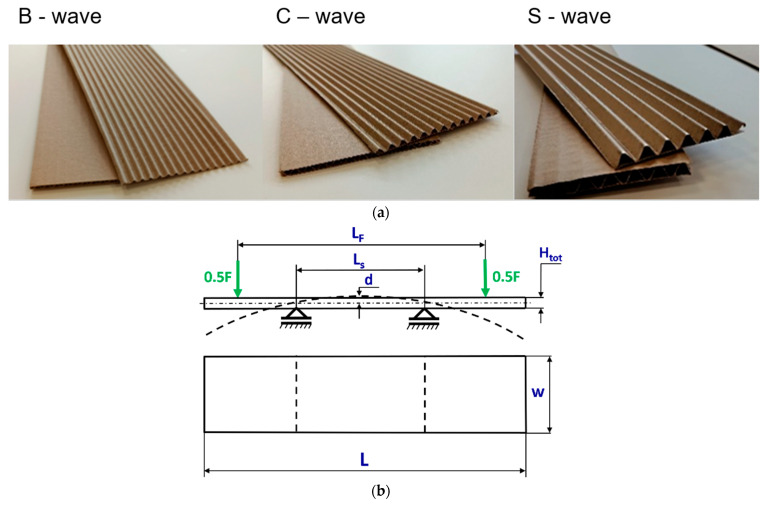
View of corrugated boards with two or three layers (**a**) and test scheme with dimensions of the sample (**b**).

The objects of study were corrugated boards with two and three layers. The tested corrugated boards were made of papers with the same fibre raw material composition. The tests were carried out for the two- or three-layer corrugated boards with B, C, and S waves shown in Figure 1a. Based on Figure 1b, the dimensions of the panel were assumed: the total length L = 500 mm, width w = 100 mm, and *H_tot_* ranged from 2.68 mm to 9.038 mm in reference to the considered model. The panel of corrugated board was subjected to four-point bending tests in the cross direction *CD*, where the distance between spans *L_s_* was 200 mm and *L_F_* (between applied forces) was 400 mm. During the measurements, a graph of the relationship between force *F* and deflection d was registered.

**Figure 2:**
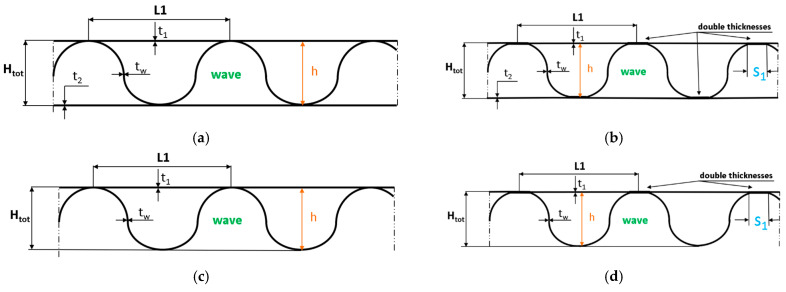
The shape and dimensions of the considered paperboard for three-layer GEOM_1 (**a**), GEOM_2 (**b**), and for two-layer GEOM_1 (**c**) and GEOM_2 (**d**).

For numerical simulation, two different models have been considered (GEOM_1 and GEOM_2) for two- or three-layer paperboard. The first of them, presented in Figure 2a,c for three- and two-layer corrugated board, respectively, relied on the purely theoretical assumption where connections between waves and flat surfaces were only linear (it corresponds to a perfect structure). The second one was assumed to be related to the real ones as presented in Figure 1a. Therefore, the connections between waves and flat surfaces were considered on some surfaces (this width was denoted *S*_1_—Figure 2b,d). Figure 2a–d shows geometric magnitudes as thicknesses of the papers *t*_1_, *t*_2_, *t_w_*, distances of wave *L*_1_, heights of wave *h*, and total height *H_tot_* describing the geometry of the analysed paperboard. Parameter *S*_1_ in dependence upon the variant was equal to between 1.28 mm and 3.44 mm.

### 2.2. Material Properties

**Figure 3:**
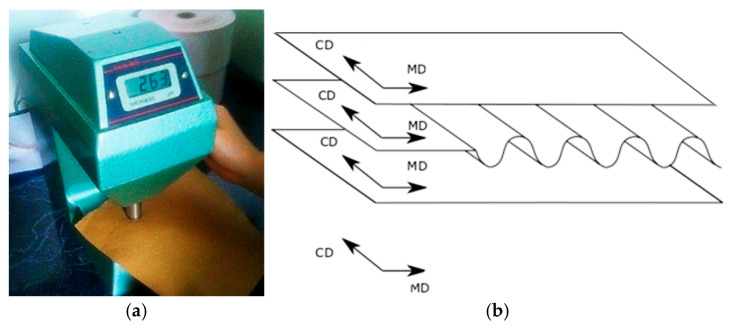
Micrometer for measuring the thicknesses (**a**) and view of orientation in case of *MD* and *CD* (**b**).

For the papers from which the corrugated boards were made, the thickness was measured using a micrometer (Figure 3a) equipped with two parallel pressure plates of 2 cm^2^ each, applying a pressure of 100 ± 10 kPa, which was exerted on the test paper by the surfaces of the plates. Twenty measurements were taken for each paper. The thickness of the papers was given as the average of the 20 measurements. This enabled the paper thickness to be measured with an accuracy of 0.001 mm (Table 1).

The material of the paper was modelled as an orthotropic one with a linear range. Young’s modulus for machine direction (*MD*) and cross direction (*CD*) of each paper were defined due to one-directional tensile tests at a constant tensile speed of 20 mm/min and a clamping length of 180 mm in accordance with the PN-EN ISO 1924-2:2010 [46]. The test sample had a standardized width of 15 mm. The measurement was carried out on a Zwick Tensile Machine model Z010 (ZwickRoell, Ulm, Germany) equipped with clamping chucks.

During the measurement, a graph of the relationship between force *F* and deformation *l* was recorded, based on which the ratio of the increase in force to the increase in elongation ∆*F*/Δ*l*, was determined as the slope coefficient of the equation of the tangent to the rectilinear section of the graph inclined at the greatest angle to the abscissa axis. The value of Young’s modulus was calculated using Equation (1).

(1) $$E=\frac{∆F\;\cdot \;l_{0}}{b_{p\;}\;\cdot t\;\cdot \;∆l}$$

where: *l*_0_—sample clamping length amounting to 180 mm, *b_p_*—paper sample width, mm, in our case 15 mm, *t*—average sample thickness, mm, Δ*F*—sample load increase (determined based on the graph), N, Δ*l*—elongation increase (determined based on the graph), mm.

The orientation of *MD* and *CD* is shown in Figure 3b. The values of moduli *E_MD_*, *E_CD_* given in Table 1 denote mean values from 10 separate measurements. Poisson’s ratios *n_MD_, n_CD_* were based on the literature; however, shear moduli *G_MD-CD_* were obtained based on the formula (0.33·*E_MD_·E_CD_*)^0.5^.

**Figure 4:**
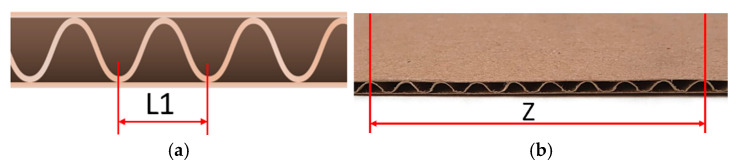
Wave distances in corrugated board (**a**), measured length (**b**).

The geometric parameters of the corrugated board were then examined. The thickness of the corrugated board, *H_tot_*, was measured using a micrometer. The micrometer is equipped with two pressure feet, with the upper foot having a diameter of 35.7 mm ± 0.3 mm and the lower foot contacting the entire area of the second wall when the micrometer points to zero. During the measurement, a pressure of 20 kPa ± 0.5 kPa was exerted on the corrugated paperboard. For each corrugated board, 20 measurements were taken, and the final result is the average value of the 20 measurements. The *L*_1_ wave distances are the distances between two adjacent wave vertices adjacent to one plane layer (Figure 4a). In order to determine the wave pitch on one of the flat sheets of corrugated cardboard, 10 wave peaks were marked, and the distance between them was measured as *Z* (Figure 4b).

The wave pitch was calculated from the formula given in Equation (2).

(2) $$L_{1}=\frac{Z}{Number\;of\;wave}\;[\mathrm{mm}]$$

The wave height *h* was calculated as the difference between the thickness of the corrugated board and the thickness of the two flat layers of paper.

Determination of the corrugation coefficient involved cutting samples of the same length *l*_1_, then delaminating them under water. The corrugated layer was stretched out and dried, then air-conditioned in air with 50% humidity and a temperature of 23 °C. The length of the corrugated layer *l*_2_ was measured. This magnitude was formulated by Equation (3).

(3) $$Corrugation\;coeffcient=\frac{l_{2}}{l_{1}}$$

The geometric parameters of the corrugated boards are summarized in Table 2.

**Figure 5:**
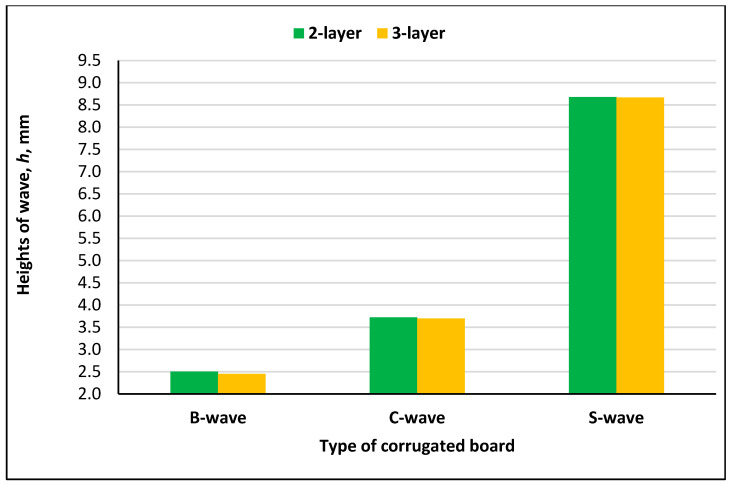
Heights of wave and difference in height for two-layer and three-layer corrugated boards.

Based on the results of measurements of geometric parameters of the tested two-layer and three-layer corrugated boards, it was observed that in all cases, the height of the wave *h* was higher for the two-layer corrugated boards than for the three-layer corrugated boards (Figure 5).

**Figure 6:**
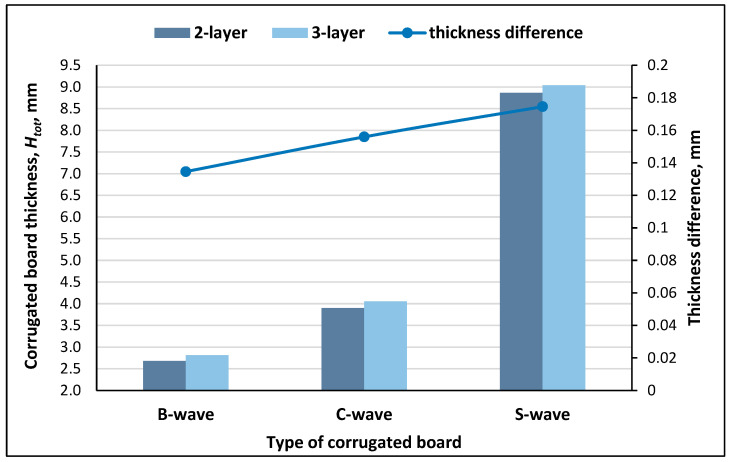
Thickness and difference in thickness for two-layer and three-layer corrugated boards.

Comparing the thickness of two-layer and three-layer corrugated boards, it was observed that the differences in the thickness of three-layer and two-layer boards increased with increasing wave height. The reason for the greater differences was probably the greater deformation of the wave with increasing wave height during the production of corrugated board at the stage of applying glue to the tops of the waves and connecting the second layer of flat paper to the wave. The smallest difference in the thickness of two-layer and three-layer corrugated paperboards was recorded for B-wave boards, the largest for S-wave boards (Figure 6).

### 2.3. Test Stand

**Figure 7:**
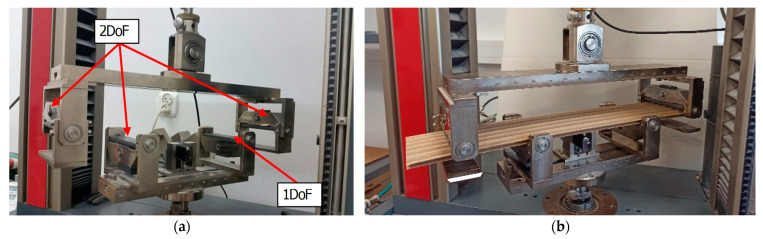
Tool for measuring bending stiffness (**a**) [32] and a sample of the corrugated board placed in the tool (**b**).

Before tests, paperboards were subjected to drying at a temperature of 40 °C. Afterwards, paperboards were conditioned based on standard PN-EN 20187:2000 (temperature 23 ± 1 °C and relative humidity 50 ± 2%) until thermodynamic equilibrium was reached [47]. Tests of bending were performed on the Zwick Tensile Machine model Z010 using a dedicated tool (Figure 7a). The tool consists of four supports. Three of them (two upper supports and one lower) have two DoF, and the fourth one has only one DoF. The load range of the machine allows them to examine a range of 0.1 N to 10 kN. The supports of the *BS* bending stiffness measuring device had a rectangular cross-section with dimensions of 3 cm × 10.3 cm. In each test, the movable jaws of the machine were moving at a speed of 10 mm/min. The bending stiffness in the cross direction *CD* of the corrugated board was investigated. The view of setting the sample in the measuring grip is presented in Figure 7b. Based on the applied standard, the bending tests relied upon a preload (5–10% range of maximum load) to determine the stiffness in bending.

### 2.4. Bending Stiffness

Bending stiffness *BS* can be treated as one of the most important coefficients used in papermaking. This magnitude is formulated by Equation (4) in accordance with Figure 1b. *BS* is expressed in units Nm. *BS* is registered in the range of 10%—40% to full loading (before large deformations occurred).

(4) $$BS=\frac{\frac{F}{2}\;\frac{\left(L_{F}-L_{S}\right)}{2}\;\cdot \;L_{s}^{2}}{8\;w\;d}=\frac{F\;{\cdot (L}_{F}-L_{S})\;\cdot \;L_{s}^{2}}{32\;w\;d}$$

**Figure 8:**
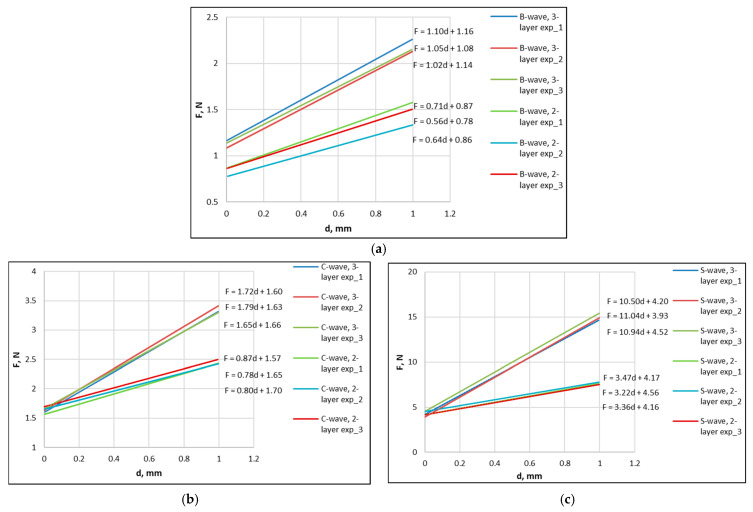
Dependence of force *F* on deflection *d* for the tested corrugated boards: (**a**) wave B, (**b**) wave C, (**c**) wave S.

Figure 8a–c compares the graphs of the relationship between force *F* and deflection *d* for two-layer and three-layer corrugated boards with B-wave, C-wave, and S-wave, respectively.

In the above graphs, it can be seen that with the increase in wave height, the difference in the values of the angle of inclination of the lines of the dependence of the force *F* on the deflection d of three-layer and two-layer corrugated boards increases, which is also evidenced by the values of the directional coefficient of the straight lines.

The results of the bending stiffness measurement in the transverse direction of *BS_CD_* are summarized in Table 3, along with the statistical processing of the results.

The obtained *BS_CD_* results prove that as the wave height and corrugated board thickness increase, the *BS_CD_* difference for the considered two-layer and three-layer corrugated paperboards increases. The *BS_CD_* value of the three-layer corrugated board with B-wave was 1.7 times higher than the *BS_CD_* obtained for two-layer paperboard with B-wave. For the three-layer corrugated board with C and S waves, the increase was much higher, equal to 2.1 times and 3.2 times, respectively.

**Figure 9:**
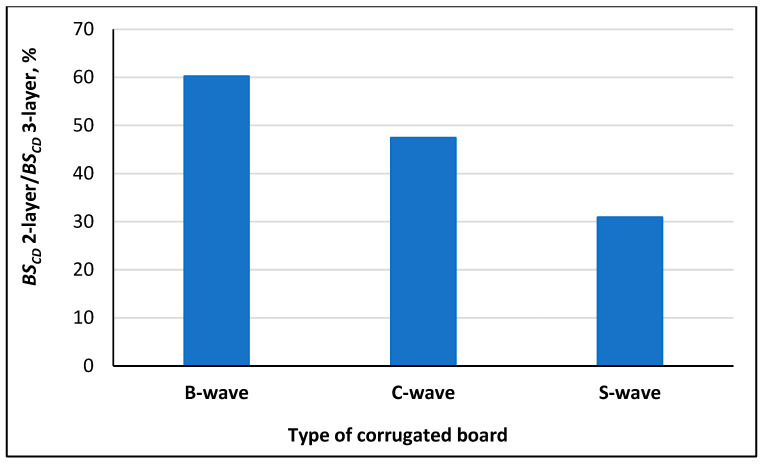
Percentage of *BS_CD_* of two-layer corrugated boards in *BS_CD_* of three-layer corrugated boards.

The percentage of the bending stiffness of two-layer corrugated board in the bending stiffness of three-layer corrugated board was calculated as the ratio of two-layer *BS_CD_* to three-layer *BS_CD_* and expressed as a percentage, and is shown in Figure 9.

Figure 9 shows a significant decrease in the proportion of *BS_CD_* of two-layer paperboards in the *BS_CD_* of three-layer paperboards as the B to S wave height increases. The percentages are 60.25% for the B-wave, 47.48% for the C-wave, and 30.94% for the S-wave, respectively.

### 2.5. FE Models

**Figure 10:**
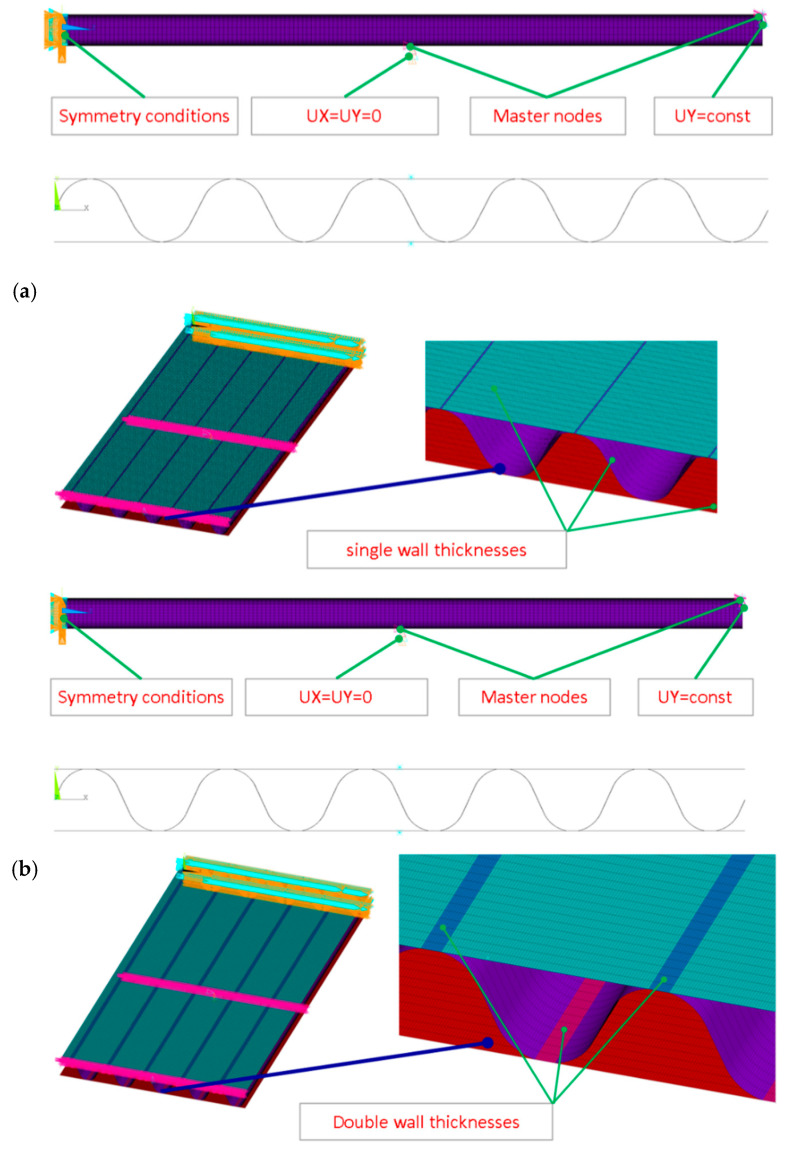
Numerical models with assumed boundary conditions: GEOM_1 (**a**) and GEOM_2 (**b**).

Numerical simulations of each model were conducted for a half of the panel (200 mm) and a width of 100 mm in Ansys^®^ software version 2025 R1 [45]. In the analysis, conditions of symmetry were set for both models (Figure 10a,b). The discrete models were elaborated by means of a 4-node 181-element matrix. Looking at the software description [45], this element can be suitable for simulations of thin or moderately thick shell structures.

The directions of orthotropy correspond to the orientation shown in the sketch (Figure 3b). In the case of GEOM_2, the touching walls between flat and corrugated paper had attributed double thicknesses (See Figure 10b). The size of the finite element was set to 0.2–0.25 mm, giving 200–400k finite elements, depending upon the number of variants. The numerical bending was accomplished by using displacement control by means of a master node. This node was tied with a group of outer nodes of the panel to bring about vertical displacement (it prevented the local deformation of the panel). The simulations were performed for large displacements of the Green–Lagrange equations. The nonlinear estimations were carried out based on the Newton–Raphson algorithm. The number of substeps was between 400 and 5000; however, the number of iterations in each substep could be as many as 2000. Such parameters ensured the convergence of calculations.

## 3. Results

This chapter compares the results obtained from the measurements with those obtained during FE simulations for all tested two- and three-layer corrugated boards and the proposed FE models.

### Stiffness Analysis

**Figure 11:**
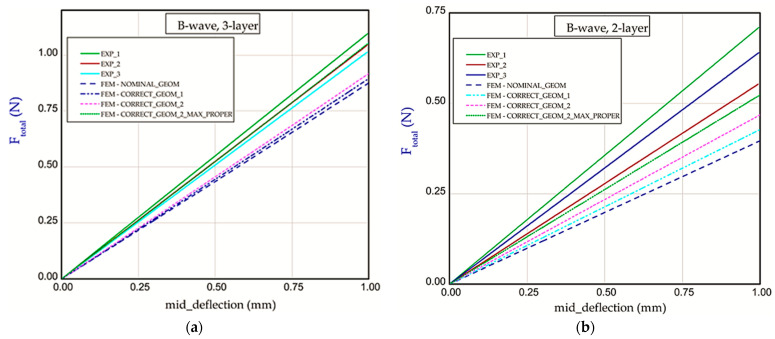
Force vs. mid-deflection for Model_1 with three layers (**a**) and for Model_1 with two layers (**b**).

**Figure 12:**
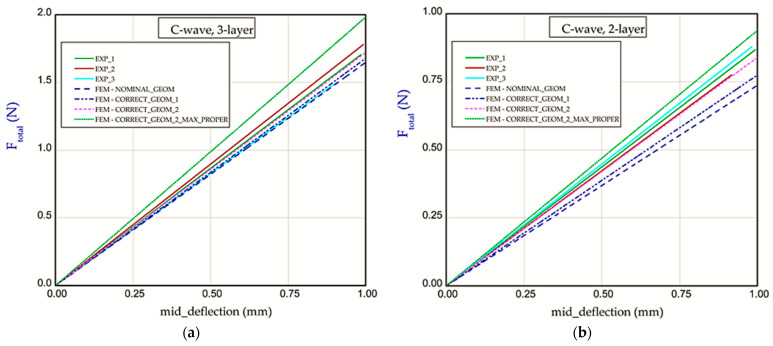
Force vs. mid-deflection for Model_2 with three layers (**a**) and for Model_2 with two layers (**b**).

**Figure 13:**
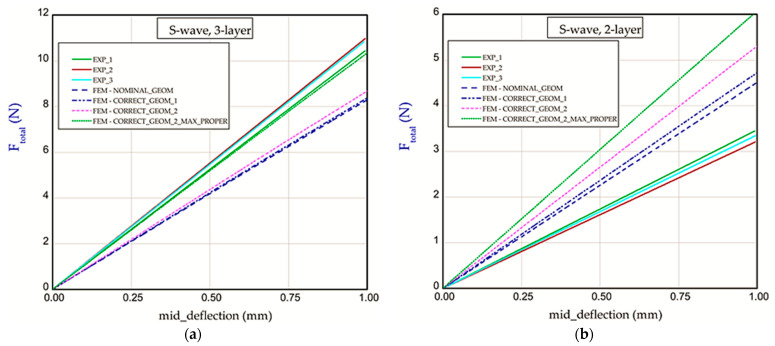
Force vs. mid-deflection for Model_3 with three layers (**a**) and for Model_3 with two layers (**b**).

This subsection includes the results of the bending of all the models. The diagrams shown are expressed in a function of bending force *F_total_* vs. mid_deflection (*d*) (Figure 11, Figure 12 and Figure 13). The experimental tests were performed for three samples of each considered model (two- or three-layer) without causing it damage. Figure 11 presents the curves of bending of Model_1 (B-wave). In this case, the thicknesses of papers applied for the corrugated paperboard are equal to *t*_1_ = *t*_2_ = 0.181 mm and *t_B_* = 0.164 mm. Numerical calculations were performed for the nominal height of the panel (NOMINAL_GEOM, then *H_tot_* = 2.814 mm). It can be noticed that the stiffness for NOMINAL_GEOM is slightly smaller than the stiffness obtained experimentally for both two-layer and three-layer corrugated paperboards. Due to increasing the extent of touching surfaces (CORRECT_GEOM_1 with *S*_1_ = 1.28 mm) to achieve a more realistic model of the panel, the stiffness grew only by a few percent, and this result is still lower than the experimental one.

Based on the results of CORRECT_GEOM_2 (with *S*_1_ = 1.65 mm), a delicately better result appeared, but it is still far away from the experimental results. Neither of the curves with corrected geometry significantly improved the stiffness. Taking into account the variant CORRECT_GEOM_2_MAX_PROPER with maximal material properties obtained during measuring, one can see comparable lines with experimental lines for the three-layer corrugated board (Figure 11a). On the other hand, in the case of the two-layer paperboard, the discrepancy between numerical and empirical results is greater (Figure 11b). Moreover, the repeatability of results in experiments is not obvious, and the range in stiffness is quite large. In contrast, calculations for C-wave (Figure 12a,b) allowed us to obtain results comparable with the experimental ones regardless of assuming a version of numerical models (CORRECT_GEOM_1 with *S*_1_ = 1.28 mm and CORRECT_GEOM_2 with *S*_1_ = 1.77 mm). In the case of variant CORRECT_GEOM_2_MAX_PROPER (Figure 12a), stiffness is higher in reference to the experiment. Based on the results for two-layer paperboard (Figure 12b), the situation is almost the same (the closest result was observed for variant CORRECT_GEOM_2).

Taking a look at the results for S-wave (Figure 13a), CORRECT_GEOM_1 with *S*_1_ = 2.85 mm and CORRECT_GEOM_2 with *S*_1_ = 3.44 mm, one can notice a comparable effect as for paperboard with B-wave. It means that numerical results gave the lowest stiffnesses in the experiment (beyond the variant with maximal properties). In turn, results for two-layer paperboard (Figure 13b) showed a reverse effect because all numerical curves turned out to have higher stiffnesses. The *BS* values calculated for all considered variants are sorted out in Table 4. Based on the results from Table 4, the stiffnesses of paperboard in many cases are comparable with the experimental values.

**Figure 14:**
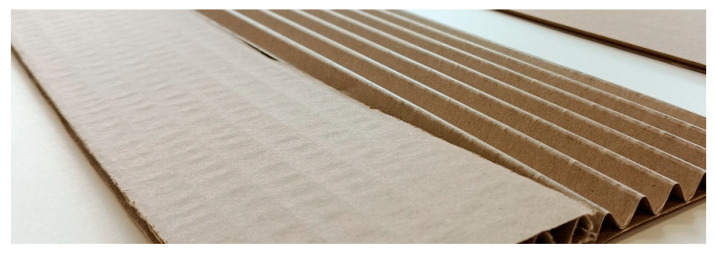
Wrinkled flat layer—defect.

Nevertheless, great discrepancies are noticed as well (e.g., S-wave, two-layer), where the numerical model in each case shows greater stiffness than in the experiment. The reason for the lower bending stiffness values of the two-layer S-wave corrugated board obtained during the measurements is probably the lack of tension in the flat corrugated board layer caused by a defect (wrinkled flat liner) (Figure 14). This defect contributes to the buckling of the flat layer under compression during the *BS_CD_* measurement of the two-layer board. The numerical variant of paperboard with C-wave turned out to be convergent with the experimental result. It can be stated that the results of numerical approaches with strict geometrical data and material properties are not always close to experimental ones. Differences can result from many points.

## 4. Conclusions

This work shows the results of the analysis of four-point bending of two-layer and three-layer corrugated board. The experimental and numerical analyses were performed for three models characterized by different heights of waves.

The assumption of nominal thicknesses in numerical models usually provides lower stiffnesses relative to the experimental ones.

Increasing the extent of touching surfaces (CORRECT_GEOM_1, CORRECT_GEOM_2) allowed for obtaining comparable stiffness based on experiment and numerical approaches. However, CORRECT_GEOM_2 with maximal properties gave even higher stiffness (C-wave, two or three layers, S-wave, two layers).

Summarizing, each numerical approach applied in the present paper does not provide an exact result with respect to the experimental results, considering various paperboard geometries (different dimensions of paperboard). This shows how many parameters (both mechanical and geometrical) can influence the final result.

Comparing three-layer corrugated boards with two-layer corrugated boards, approximately 30–60% higher stiffness values were obtained for three-layer paperboards. However, a decidedly different situation occurred for three-layer boards (for larger paperboards among the analysed ones), where differences in stiffness of two-layer and three-layer boards were significantly greater (by about 3 times). In general, these differences could result from the fact that moments of cross-section of two-layer corrugated boards were smaller (in contrast to three-layer paperboard). Another reason for the smaller general stiffness was the lack of outer flat layer bonding to the peaks of the wave; therefore, the stiffness of the wave in this range significantly decreases. This could affect the general stiffness of paperboards.

## Figures and Tables

**Table 1:** Mechanical material data and thicknesses.

Model Number	Number of Layers	Thickness [mm]	*E_MD_* [GPa]	*E_CD_* [GPa]	*n_MD_* [-]	*n_CD_* [-]	*G_MD-CD_* [GPa]
Model_1 (2-layer)	1-flat (*t*_1_)	0.181	4.86	2.11	0.130	0.3	1.84
	B-wave (*t_B_*)	0.164	4.85	2.04	0.126	0.3	1.81
Model_1 (3-layer)	1-flat (*t*_1_)	0.181	4.86	2.11	0.130	0.3	1.84
	B-wave (*t_B_*)	0.164	4.85	2.04	0.126	0.3	1.81
	3-flat (*t*_2_)	0.181	4.86	2.11	0.130	0.3	1.84
Model_2 (2-layer)	1-flat (*t*_1_)	0.181	4.86	2.11	0.130	0.3	1.84
	C-wave (*t*_C_)	0.164	4.85	2.04	0.126	0.3	1.81
Model_2 (3-layer)	1-flat (*t*_1_)	0.181	4.86	2.11	0.130	0.3	1.84
	C-wave (*t*_C_)	0.164	4.85	2.04	0.126	0.3	1.81
	3-flat (*t*_2_)	0.181	4.86	2.11	0.130	0.3	1.84
Model_3 (2-layer)	1-flat (*t*_1_)	0.186	5.47	1.78	0.098	0.3	1.79
	S-wave (*t*_S_)	0.205	5.16	2.04	0.119	0.3	1.86
Model_3 (3-layer)	1-flat (*t*_1_)	0.186	5.47	1.78	0.098	0.3	1.79
	S-wave (*t*_S_)	0.205	5.16	2.04	0.119	0.3	1.86
	3-flat (*t*_2_)	0.186	5.47	1.78	0.098	0.3	1.79

**Table 2:** Geometrical parameters of corrugated boards.

Paperboard Type	*h* [mm]	*L*_1_ [mm]	*H_tot_* [mm]	*Corrugation Coefficient*
B-wave, 2-layer	2.498	6.556	2.678	1.3
B-wave, 3-layer	2.452	6.556	2.813	1.3
C-wave, 2-layer	3.719	8	3.899	1.42
C-wave, 3-layer	3.694	8	4.055	1.42
S-wave, 2-layer	8.677	15	8.863	1.62
S-wave, 3-layer	8.666	15	9.038	1.62

**Table 3:** *BS_CD_* measurement results.

Paperboard Type	*BS_CD_* Average Value, Nm	*SD*, Nm	Max. *BS_CD_*, Nm	Min. *BS_CD_*, Nm
B-wave, 2-layer	1.59	0.19	1.78	1.40
B-wave, 3-layer	2.64	0.10	2.75	2.55
C-wave, 2-layer	2.04	0.12	2.18	1.95
C-wave, 3-layer	4.30	0.18	4.48	4.13
S-wave, 2-layer	8.38	0.31	8.68	8.05
S-wave, 3-layer	27.07	0.72	27.60	26.25

**Table 4:** *BS_CD_* for all considered models.

Variant	EXP				FEM NOMINAL_GEOM [Nm]	FEM CORRECT_GEOM_1 [Nm]	FEM CORRECT _GEOM_2 [Nm]	FEM CORRECT _GEOM_2 MAX_PROP [Nm]	FEM NOMINALGEOM Decrease (−)/ Increase (+) with Respect Mean Value [%]	FEM CORRECT GEMO 1 Decrease (−)/ Increase (+) with Respect Mean Value [%]	FEM CORRECT GEMO 2 Decrease (−)/ Increase (+) with Respect Mean Value [%]	FEM CORRECT_GEMO 2 MAX_PROP Decrease (−)/ Increase (+) with Respect Mean Value [%]
	1 [Nm]	2 [Nm]	3 [Nm]	Mean Value [Nm]								
B-wave, 3-layer	2.75	2.63	2.55	2.64	2.19	2.24	2.30	2.64	17.14	15.22	12.85	0.20
B-wave, 2-layer	1.78	1.40	1.60	1.59	0.99	1.07	1.17	1.31	37.75	32.86	26.59	17.85
C-wave, 3-layer	4.30	4.48	4.13	4.30	4.12	4.20	4.35	4.96	4.12	2.39	−1.26	−15.30
C-wave, 2-layer	2.18	1.95	2.00	2.04	1.84	1.94	2.09	2.35	9.68	5.11	−2.59	−14.93
S-wave, 3-layer	26.25	27.60	27.35	27.07	20.69	20.88	21.64	25.79	23.56	22.84	20.04	4.72
S-wave, 2-layer	8.68	8.05	8.40	8.38	11.27	11.81	13.26	15.19	−34.60	−41.01	−58.30	−81.42

## Data Availability

The original contributions presented in the study are included in the article, further inquiries can be directed to the corresponding author.
